# Impact of collaborative pharmaceutical care on in-patients’ medication safety: study protocol for a stepped wedge cluster randomized trial (MEDREV study)

**DOI:** 10.1186/s13063-017-2412-7

**Published:** 2018-01-08

**Authors:** Géraldine Leguelinel-Blache, Christel Castelli, Clarisse Roux-Marson, Sophie Bouvet, Sandrine Andrieu, Philippe Cestac, Rémy Collomp, Paul Landais, Bertrice Loulière, Christelle Mouchoux, Rémi Varin, Benoit Allenet, MEDREV Working Group, Pierrick Bedouch, Jean-Marie Kinowski

**Affiliations:** 1 Department of Pharmacy, Nîmes University Hospital, Nîmes, France; 2 UPRES EA 2415, Laboratory of Biostatistics, Epidemiology, Clinical Research and Health Economics, Clinical Research University Institute, Montpellier University, Montpellier, France; 3 Department of Biostatistics, Epidemiology, Clinical Research and Health Economics, Nîmes University Hospital, Nîmes, France; 4 Epidemiology and Public Health Research Unit, INSERM 1027, Toulouse, France; 5 Department of Pharmacy, Nice University Hospital, Nice, France; 6 Observatory of Medicine, Medical Devices and Therapeutic Innovation, Regional Health Agency, Bordeaux, France; 7 Research Clinic Centre (CRC) - VCF (Aging – Brain - Frailty), Charpennes Hospital, University Hospital of Lyon, Villeurbanne, France; 8 INSERM U1234, PANTHER unit, Faculty of Medicine and Pharmacy, University of Rouen, Rouen, 76183 France; 9 TIMC-IMAG UMR CNRS 5525, Grenoble Alpes University, Grenoble, France

**Keywords:** Stepped wedge study, Medication reconciliation, Medication review, Pharmaceutical care, Hospital pharmacist, Drug-related problem, Drug safety

## Abstract

**Background:**

Clinical pharmaceutical care has long played an important role in the improvement of healthcare safety. Pharmaceutical care is a collaborative care approach, implicating all the actors of the medication circuit in order to prevent and correct drug-related problems that can lead to adverse drug events. The collaborative pharmaceutical care performed during patients’ hospitalization requires two mutually reinforcing activities: medication reconciliation and medication review. Until now, the impact of the association of these two activities has not been clearly studied.

**Methods:**

This is a multicentric stepped wedge randomized study involving six care units from six French University Hospitals (each unit corresponding to a cluster) over seven consecutive 14-day periods. Each hospital unit will start with a control period and switch to an experimental period after a randomized number of 14-day periods. Patients aged at least 65 years hospitalized in one of the participating care units and having given their consent to be called for a 30-day and 90-day follow-up can be enrolled. For each 14-day period, 15 patients will be recruited in each care unit to obtain a total of 630 patients enrolled in all centers. Patients with a hospital stay of more than 21 days will be excluded. During the control period, there will be no clinical pharmacist in the care unit, whereas during the experimental period a clinical pharmacist will perform medication reconciliation and review with the healthcare team. The primary outcome will assess the impact of collaborative pharmaceutical care on preventable medication error rate. The secondary outcomes will evaluate the clinical impact of the strategy, the acceptance rate of pharmaceutical interventions, the induced and avoided costs of the strategy (cost-consequence analysis), and the healthcare team’s satisfaction.

**Discussion:**

This study will assess the impact of collaborative pharmaceutical care associating medication reconciliation and review at patient admission to hospital in terms of preventable medication error rate and costs. This activity will prevent and correct medication errors arising earlier in the hospitalization.

**Trial registration:**

ClinicalTrials.gov, NCT02598115. Registered on 4 November 2015.

**Electronic supplementary material:**

The online version of this article (doi:10.1186/s13063-017-2412-7) contains supplementary material, which is available to authorized users.

## Background

Drug-related problems (DRP) are a major concern in terms of public health because they have a serious clinical and economic impact on healthcare systems. A French multicentric study [1] reported that adverse drug events (ADE) were the second most common complication during hospitalization and were responsible for around 130,000 hospitalizations and 10,000 deaths a year in France. Apretna et al. estimated that each ADE cost > €5000 on average [2], yet around half of the severe ADEs are considered to be preventable [1]. Medication errors that can lead to ADE mainly occur at transition points in the patient’s healthcare pathway, mostly due to defects in the transmission of information at the prescription and the administration steps [3]. Also, 47–67% of patients have at least one error or discrepancy between the medications prescribed in the community and in the hospital. These errors are responsible for severe ADEs in 18–59% of cases [3–6]. Elderly patients are frequently frail and are at particularly high risk of DRPs. Indeed, 32–46% of hospitalized elderly patients experience at least one ADE [7, 8], of which 28% were deemed to be preventable [9].

For years, pharmaceutical care has been a key strategy to improve healthcare safety. Pharmaceutical care is a collaborative care approach which implies all the actors of the medication circuit in order to prevent and correct DRPs that can lead to ADEs. The collaborative pharmaceutical care performed during patients’ hospitalization requires two complementary activities: medication reconciliation and medication review. First, medication reconciliation is a collaborative process requiring the acquisition of the best possible medication history (BPMH) and its comparison against the hospital medication order [5, 10]. Studies have shown that medication reconciliation had a potential clinical impact by decreasing the rate of hospitalized patients with at least one unintended medication discrepancy (UMD) [10, 11]. This activity could be optimized by a clinical pharmacist based in care units [12]. A few studies have demonstrated that medication reconciliation can reduce readmissions [10, 13], whereas 10–24% of UMDs would lead to severe harm or would be life-threatening [11, 14]. Second, medication review is a multidisciplinary analysis of medication order initiated by the clinical pharmacist [10, 15, 16] that consists of optimizing the medication treatment by improving medication adherence, compliance with the recommendations, and decreasing DRPs (e.g. detection of potential inappropriate medications in elderly [PIM], non-treated therapeutic indications, better matching between medication and therapeutic indication, etc.). Medication review is supported by international standardized tools such as STOPP and START criteria [17, 18], Beers Criteria [19], and the Medication Appropriateness Index [20]. Medication reviews have highlighted PIM representing 16–71% of medications prescribed at hospital admission [21–23]. Moreover, PIM can lead to preventable ADEs [24]. Studies have shown that 38–79% of the patients benefiting from a medication review initiated by a pharmacist had at least one medication change made by the physician in the order [22, 25, 26]. Some studies have shown an impact of medication review on readmission induced by DRPs [25, 27].

However, very few studies have explored the health economics impact of medication reconciliation and medication review in European health systems. Malet-Larrea et al. showed that medication review associated with a clinical pharmacist follow-up led to an estimated saving of €273 per patient-year [28]. Moreover, a structured pharmacist review of medication supported by computerized clinical decision support software demonstrated a decrease of €807 in mean healthcare costs [29]. To the best of our knowledge, the economic impact of the association of medication reconciliation and review in a collaborative pharmaceutical care process has never before been studied.

The primary outcome of our study will assess the impact of collaborative pharmaceutical care on preventable medication error rate. The secondary outcomes will evaluate the clinical impact of the strategy, the acceptance rate of pharmaceutical interventions, the induced and avoided costs of the strategy (cost-consequences analysis), and the healthcare team’s satisfaction.

## Methods/design

A scientific committee will oversee the methodology of the study and ensure that the research organization is relevant to ensure the quality of the collection. A meeting of the Scientific Committee of the MEDREV study will be organized when the project is accepted, before the start of the study, and when the results are exploited.

### Design

**Fig. 1:**
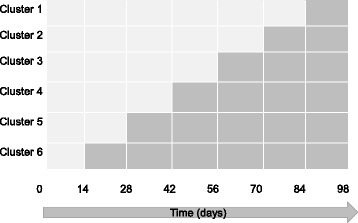
MEDREV study design

A multicentric stepped wedge cluster randomized trial is proposed because randomization of patients was not possible in this study due to the high risk of contamination bias. Indeed, once the clinical pharmacist arrives in the care unit, the collaborative pharmaceutical care will lead to change in medical practices for all the patients, including the control group. Stepped wedge randomized trial designs involve sequential roll-out of an intervention to participants over a number of time periods. By the end of the study, all participants will have received the intervention, although the order in which participants receive the intervention is determined at random. The clusters will be hospital units. Each hospital unit will start with a control period and switch to an interventional period. The study design will be composed of seven consecutive 14-day periods (Fig. 1). Every 14-day period, one further hospital unit will switch to the interventional period until all the units are in the interventional period in the final 14-day period. The randomization by the project methodologist will define the point that each hospital unit will switch from the control to the interventional periods (Table 1).

**Table 1:** Centers, units, participants in the control period, and switch period

Hospital	Unit	Participants in the control period	Intervention starting period
CHU Rouen	Diabetes, endocrinology and metabolic diseases	Pharmacy students	Period 2
CHU Toulouse	Cardiology	Pharmacy students	Period 3
CHU Nice	Geriatric internal medicine	Pharmacy technicians	Period 4
CHU Grenoble	Geriatric acute medicine	Pharmacy students	Period 5
CHU Strasbourg	Internal medicine	Pharmacy technicians	Period 6
CHU Nîmes	Medical emergency	Pharmacy technicians	Period 7

*CHU* University Hospital Center

A randomized cluster trial would be difficult to conduct because the cluster number would be high and half of them would not benefit from the intervention.

### Setting and population

Six French University Hospitals which benefit from a strong expertise in clinical pharmacy and are dispersed across the country have agreed to participate (Table 1). Each center selected a short-term care unit in a medical specialty which welcomes the elderly. Only units in which there was no clinical pharmacist were eligible. The medical heads of all the units selected agreed to participate in the study.

All healthcare participants volunteered to participate in the study. In each center, two pharmacy students or two pharmacy technicians will participate in the control period (Table 1); two senior clinical pharmacists will participate in the interventional period. In each hospital center, the same clinical pharmacists will detect medication errors in both periods. All the participants benefited from a specific centralized training in order to harmonize their practices in each period. This training was performed over three days, the week before the study started. After data collection, controls will be carried out to ensure that pharmaceutical interventions have not been misclassified.

Patients aged at least 65 years, hospitalized in one of the participating care units, and having given their agreement to be called for a 30-day and 90-day follow-up could be enrolled. In each 14-day period, 15 patients will be recruited in each care unit no more than 24 h or 48 h after admission, respectively, in the week or weekend/public holiday. Finally, a total of 630 patients will be enrolled in the study. We will exclude patients with a hospital stay > 21 days, as there is a risk of there being too many medication modifications during the hospital stay.

**Fig. 2:**
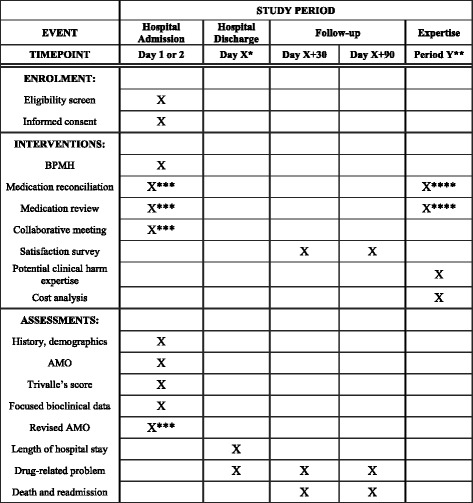
Standard Protocol Items: Recommendation for Interventional Trials (SPIRIT) figure. * X is 1–21 days. ** Y is a period after the end of the post-discharge follow-up. *** Interventional period. **** Observational period. AMO admission medication order, BPMH best possible medication history

The study procedures and assessments are outlined in the Standard Protocol Items: Recommendation for Interventional Trials (SPIRIT) checklist (see Additional file 1) and SPIRIT Figure (Fig. 2).

### Outcomes

The primary outcome will assess the rate of patients having at least one preventable medication error (e.g. omission, wrong dose, wrong route of administration, etc.) on admission medication order (AMO). The primary outcome will be evaluated by clinical pharmacists retrospectively in the control period and prospectively in the interventional period.

Among the secondary outcomes, the potential clinical impact of each error detected will be assessed by a hospital physician and two clinical pharmacists (different from the investigators) by using a decision algorithm [30] adapted from the NCC MERP’s Index [31] and employing the high alert risk medication list of the Institute of Safe Medication Practices [32] and the North Carolina Narrow Therapeutic Index [33]. All of the experts, who are seniors specialized in geriatrics, will blindly perform the analysis, i.e. without knowing the hospital center concerned, after completion of patient recruitment for all the centers. The potential clinical impact will also be evaluated by the rate of patients at high risk of ADEs according to Trivalle’s score [9, 34].

Other secondary outcomes will be: (1) the acceptance rate of pharmaceutical interventions during the interventional period; (2) the readmission and mortality rates 30 and 90 days after discharge; (3) the length of hospital stays; (4) the satisfaction of care providers involved in the collaborative pharmaceutical care (assessed by postal or electronic mail after the end of the 90-day follow-up); and (5) the induced and avoided costs of the strategy. For this last outcome, we will estimate the induced costs by measuring the time spent performing the collaborative pharmaceutical care. We will also assess the avoided costs by estimating the healthcare consumptions dues to medication errors by using the results of a French national study concerning ADE occurrence and cost [1] and/or by using an expert concertation via a DELPHI method.

### Intervention

**Fig. 3:**
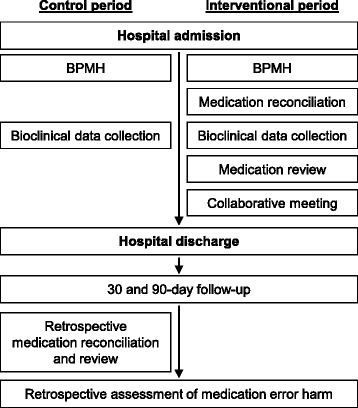
MEDREV study - flow of the intervention. BPMH, best possible medication history

The flow of the intervention is outlined in Fig. 3. During the control period, there will be no clinical pharmacist in the care unit. The hospital physician will write the AMO, then a pharmacy technician or a pharmacy student will perform the best possible medication history (BPMH) according to the SOP MED’REC [35] and collect all the relevant bioclinical data to perform prescription review. No information will be transmitted to the healthcare team except in life-threatening emergencies. A clinical study technician from the promoter center will call all the patients at 30 ± 10 and 90 ± 10 days after their hospital discharge to determine whether they had died or been re-hospitalized. If they report that they had been re-hospitalized, a pharmacy resident from the promoter center will call them again to investigate if the cause of the hospitalization is due to medication regimen. After the follow-up, the medication reconciliation and review will be retrospectively conducted by the clinical pharmacists who have participated in the interventional period in each investigator center. Finally, experts will retrospectively assess the potential clinical harm of each medication error detected.

In the interventional period, a senior clinical pharmacist based in the care unit will perform medication reconciliation by comparing the BPMH to the AMO and notifying the prescriber of any possible discrepancies. He/She will collect all relevant bioclinical data and perform medication review of the AMO by using the STOPP and START tools [18], the French list of potentially inappropriate medications in elderly [36], and the PAPA guide about medication prescription adapted to the elderly, published by the French Society of Geriatric and Gerontology [37]. The clinical pharmacist will have a collaborative meeting with both the prescriber and the nurse in order to notify any possible medication errors and suggest any proposals to optimize the AMO according to the medical history, the clinical status, and the therapeutic adherence etc. (e.g. change of galenic form due to swallowing problem, dose adjustment to renal function, etc.). After the collaborative meeting, the clinical pharmacist will check whether the prescriber has accepted his/her suggestion(s) and modified the AMO. All the pharmaceutical interventions, i.e. the medication errors detected and the pharmaceutical suggestions of order modification, will be collected and characterized in a standardized form according to the French Society of Clinical Pharmacy [38]. The post-discharge follow-up and the retrospective assessment of the potential harm of each error will be performed as in the control period. At the end of the study, a satisfaction survey will be sent to all the care providers involved in the collaborative pharmaceutical care.

### Limitations and potential bias

Due to the retrospective nature of the pharmaceutical evaluation during the control period, an information bias may lead clinical pharmacists not to notify a pharmaceutical intervention or, on the contrary, to carry out an irrelevant pharmaceutical intervention. Although this bias cannot be controlled by the prescribing physician's opinion because of the retrospective mode, the relevance of these interventions will be evaluated by experts who will rate clinical criticality. In addition, in order to avoid contamination bias, the retrospective assessment will not start until after the end of the control period in each hospital.

Telephone calls to collect deaths and potential re-hospitalizations are also limited. They will be carried out by a semi-directive interview but will remain based on the patient’s statement, which may omit intentionally or unintentionally to communicate some information. It may therefore be subject to a potential information bias that should be comparable between the two periods. It should be noted, however, that the reason for hospitalization will be collected in an exploratory manner as part of a secondary criterion.

### Blinding

Due to the nature of the intervention, blinding will be not possible for patients and care providers. Therefore, this study is fully open, without reliable blinding. The only blinded outcome is a secondary one: the expert committee in charge of assessing the potential clinical impact of the medication errors detected will not know the period and the center concerned by each medication error detected.

### Sample size calculation

A stepped wedge trial was designed according to a validated methodology [39]. A previous study showed that the rate of patients with at least one medication error in the AMO was 46% and 2% for the control and experimental strategies, respectively [11]. In order to make more conservative assumptions, we decreased the expected difference from 0.44 to 0.30. The variance of the difference deducted was 0.20. The intra-class correlation coefficient was unknown but was assumed equal to 0.05 in the most conservative case. In each 14-day period, each investigator hospital center will recruit 15 patients, i.e. a total of 630 patients for the seven periods and the six hospital centers. These assumptions led to a statistical power estimated at 99% [39]. The required power was maximal in order to potentially perform subgroup analyses when justified.

### Monitoring

A monitoring committee will meet after the start of the study, every 15 days for the first month, and then every month. A clinical research associate delegated by the promoter will monitor the study in accordance with the regulations in force. It will be responsible for monitoring the rate of inclusions and alerting to possible deviations from the protocol. Bi-monthly newsletters will be sent to all study participants.

The investigator shall inform the vigilance unit at Nîmes University Hospital of any expected or unexpected serious adverse events (SAE) and any new events that may affect the safety of persons subject to research as soon as they become aware of them. Reports of expected and unexpected SAEs are made by completing a dedicated report form and sending it by fax or e-mail.

### Data management

Only persons who will be involved in the research project and identified will have access to the data entry software: OpenClinica. The data entry in the electronic case report form (e-CRF) will be controlled and formatted to prevent the entry of data out of bounds or outliers. In the event of an input change, traceability and activity tracking is ensured. An electronic signature committing the responsibility of the investigator of each center will allow the validation of the visit and the e-CRF. This software is hosted on a website within Nîmes University Hospital. Access to this application is secured and is via http://www.bespim.fr with a login and password. The data collected through this software is backed up daily on a secure network. The network is connected to the Internet and access is protected by a firewall.

The clinical data from the study will be stored on a specific server directory. Only network administrators and authorized persons in the Department of Biostatistics, Epidemiology, Clinical Research, and Health Economics may have access to this directory.

### Statistical analysis

Descriptive statistics will be reported as counts and percentages for categorical variables, means and standard deviations for continuous variables with normal distribution, and median and quartiles for others. Comparisons of baseline characteristics and of putative risk factors between the two periods will be performed. The primary outcome will assess the rate of patients having at least one preventable medication error through a mixed effect logistic regression model [40]. Let Y_ij_ be the *j*th observation (j = 1…m_i_) in the *i*th cluster (i = 1,2,…,K). The intervention variable is X_*i*_. The logistic mixed effect model is

$$ \mathrm{Logit}\left({\mathrm{p}}_{\mathrm{i}\mathrm{j}}\right)={\upbeta}_0+{\upbeta \mathrm{X}}_{\mathrm{i}}+{\mathrm{Z}}_{\mathrm{i}} $$

Where Z_i_ is the random effect for cluster *i*, normally distributed with mean 0 and variance τ^2^, X_i_ is the group indicator. Therefore, the rate of patients having at least one preventable medication will be modeled as:

$$ {\mathrm{p}}_{\mathrm{i}\mathrm{j}}=\mathrm{E}\left({\mathrm{Y}}_{\mathrm{i}\mathrm{j}}|{\mathrm{X}}_{\mathrm{i}},{\mathrm{Z}}_{\mathrm{i}}\right) $$

The other effects of putative predictors of the differences in medication adherence between patients from the control and the interventional periods will be evaluated. The secondary outcomes will be evaluated by descriptive and classical statistics: the acceptance rate of pharmaceutical interventions during the interventional period will be estimated (%) only in interventional period; the rate of readmission;, mortality at 30 and 90 days will be estimated and compared using a Chi-square test; the length of stay will be estimated and compared using a non-parametric test; and the descriptive statistics will be used to describe the results or the satisfaction of care providers.

The economic study proposed is in the form of a cost-consequence one, using the decision tree methodology. The decision tree is a decision support tool that uses a tree-like graph or model of decisions and their possible consequences, including chance event outcomes and/or resource costs. Each node of the tree is associated to a probability and each trajectory is associated to a leaf and a cost. A stochastic analysis will be performed. All probabilities will be associated to a beta distribution and 10,000 Monte Carlo simulations will estimate the mean cost and the associated confidence interval of each group (without vs with pharmacist). The robustness of the results will be checked with the sensitivity analysis. This will be done by performing a second estimation including a new set of parameters in the decision tree, in order to consider the uncertainty due to the sample, specifically on event probabilities and individual costs induced. If the results of global cost are unchanged, then the robustness could be deduced. The Tornado diagram will be used to evaluate and sort the most sensitive parameter.

The Department of Biostatistics, Epidemiology, Public Health, and Health Economics of Nîmes Hospital Center will perform the statistical analysis using R.4.0. (R Development Core Team [2017], R Foundation for Statistical Computing, Vienna, Austria; https://www.r-project.org) and TreeAge Pro 2017 (TreeAge Software, Williamstown, MA, USA; https://www.treeage.com).

### Dissemination

The scientific committee will be responsible of the publications reporting the results of the study.

## Discussion

This trial will investigate the impact of the collaborative pharmaceutical care on preventing and correcting inpatients’ medication errors and costs.

The patient recruitment is a critical parameter to guarantee the study feasibility. The care units participating in the study have been chosen for having more than one eligible patient admission a day. Two-thirds of them are not specialized in the elderly whereas some of them are geriatric specialized units. All are medical units with a large number of beds (more than 15) and a mean length of stay of 2–5 days. In order to be maximally efficient, the intervention will need to start as soon as possible after patient admission. The patient interview during the medication reconciliation process will take around 15 min.

In order to standardize the collaborative pharmaceutical care, the scientific committee has created a specific training program for the students and technicians involved in the control period and for the senior clinical pharmacists involved in the interventional period. This program was elaborated by expert clinical pharmacists who are members of the French Society of Clinical Pharmacy. To ensure the performance of the intervention, they have written quality forms (i.e. checklist process and worksheets). To harmonize the data collection, phone interviews will be centrally performed by two officers.

The next step of this phase III study, if the results lead to effectiveness of the collaborative pharmaceutical care, is to assess the implementation and to determine whether others can reliably replicate the intervention and results in uncontrolled settings over the long term.

This study has the support of the French Society of Clinical Pharmacy which is expected to write recommendations and promote the implementation of the collaborative pharmaceutical care in all French health facilities in order to improve medication safety.

### Trial status

Currently, six hospital centers have been recruited and 622 patients enrolled since 19 September 2016.

## Additional file

Additional file 1: SPIRIT 2013 Checklist: Recommended items to address in a clinical trial protocol and related documents*. (DOCX 48 kb)

## Abbreviations

ADE: Adverse drug events
AMO: Admission medication order
BPMH: Best possible medication history
CCTIRS: French advisory committee on processing information in health research
CNIL: French data protection committee
CPP: Committee for the protection of individuals
DRP: Drug-related problem
NCC MERP: United States’ National Coordination Council for Medication Errors Reporting and Prevention
PAPA: Medication prescription adapted to elderly
PIM: Potential inappropriate medication in elderly
PREPS: Research program on the performance of care system
SFPC: French Society of Clinical Pharmacy
SOP MED’REC: Standard operating protocol of medication reconciliation
START: Screening Tool to Alert doctors to Right Treatment
STOPP: Screening Tool of Older Persons’ potentially inappropriate Prescriptions
UMD: Unintended medication discrepancy
